# Linking habitat preferences and fitness across scales for a relict bird species of the southern Andes

**DOI:** 10.1038/s41598-025-93594-1

**Published:** 2025-10-06

**Authors:** Tomás A. Altamirano, Fernando J. Novoa, Zoltan Von Bernath, Alejandra Vermehren, Kathy Martin, Rocío Jara, Edwin R. Price, Ricardo Rozzi, José Tomás Ibarra

**Affiliations:** 1 Austral Mountain Conservation and Research (CIMA) Laboratory, Center for Local Development (CEDEL), Villarrica Campus, Pontificia Universidad Católica de Chile, Villarrica, Chile; 2 Fundación Mar Adentro, Pucón, Chile; 3 Department of Forest and Conservation Sciences, University of British Columbia, Vancouver, BC Canada; 4 Cape Horn International Center for Global Change Studies and Biocultural Conservation (CHIC), Universidad de Magallanes, Puerto Williams, Chile; 5 Center for Local Development (CEDEL), ECOS (Ecosystem - Complexity - Society) Co-Laboratory, Villarrica Campus, Pontificia Universidad Católica de Chile, Villarrica, Chile; 6 Environment and Climate Change Canada, Pacific Wildlife Research Centre, Vancouver, BC Canada; 7 Green Godwit Consulting LLC, Cleveland, OH 44120 USA; 8 Sub-Antarctic Biocultural Conservation Program, Department of Philosophy and Religion, Department of Biological Sciences, University of North Texas, Denton, TX USA; 9 Cary Institute of Ecosystem Studies, Millbrook, NY USA; 10 Department of Ecosystems and the Environment, Faculty of Agriculture and Natural Systems, Center of Applied Ecology and Sustainability (CAPES) & Center for Intercultural and Indigenous Research (CIIR), Pontificia Universidad Católica de Chile, Santiago, Chile

**Keywords:** Biodiversity, Conservation biology, Forest ecology

## Abstract

Animals select their habitats from available resources in a way that should maximize fitness, and thus habitat preferences are generally predicted to be adaptive. However, there may be a mismatch between habitat preferences and fitness due to factors such as limited availability or disturbance of nesting habitats. In this study, we examine whether preferred nesting habitat attributes are linked to fitness (nest survival and number of fledglings) of the White-throated Treerunner (*Pygarrhichas albogularis*), an obligate excavator and tree cavity nester, across four spatial scales: (1) cavity -for fitness influence only-, (2) nest-tree, (3) forest-stand, and (4) landscape. During eight breeding seasons (October to February), between 2010 and 2018, we found and monitored 65 Treerunner nests in Andean temperate forests, Chile. We obtained four main results. First, we found a multiscale response for both habitat preferences and fitness: variables at both nest-tree and landscape scales were the most influential for nesting habitat preferences, while variables at both cavity and nest-tree scales were the most influential for fitness. Second, the probability that a given habitat is used for nesting increased with larger trees, advanced tree decay classes, and forest cover. Third, nest survival was positively related with cavity entrance diameter, height, and distance from the forest edge. Fourth, the number of fledglings increased with south-oriented cavities and decay class, excepting for old dead trees where the breeding outcomes decreased. Combined, our results suggest a general match between habitat preferences and fitness, with a mismatch occurring with trees in advanced decay. The fact that the match occurs in areas with live unhealthy trees and recently dead trees, and a high forest cover, highlight the importance of (a) old-growth forests, as they comprise the best integration of multiscale habitat attributes for this species, and (b) maintaining the continuity of forest cover together with both live unhealthy and recently dead trees in managed and/or second-growth forests.

**Supplementary Information:**

The online version contains supplementary material available at 10.1038/s41598-025-93594-1.

## Introduction

Animals select their habitats from available resources in a way that should maximize fitness^1^. Thus, it is expected that habitat attributes associated with habitat selection will also be those most strongly linked to fitness^2^. Habitat preferences (i.e. the final pattern of habitat used with respect to its availability) will generally be adaptive, under the pressures of natural selection, if a species obtains maximum fitness^3,4^. However, there may be a mismatch between habitat preferences and fitness due to factors such as temporal changes in habitat features after territory establishment^5^ and rapid disturbance of reproductive habitats^6,7^.

Habitat attributes influence fitness via the costs (e.g. predation risk) and benefits (e.g. food availability) of habitat preferences^8^. There is evidence that avian habitat preferences are scale-dependent and hierarchical phenomena^9^. Scales at which habitat selection may occur span from microsites (e.g. tree scale) to larger areas selected for nesting and/or foraging within the home range of a species^10^. Thus, differences between nesting and available sites reported for excavators (i.e. species that excavate their nesting cavities in trees or other substrates) may occur because there are multiple scales operating in nesting site selection processes, from fine to coarse scales^11,12^. Excavators assess, for example, the tradeoff between a secure nesting substrate for excavation (e.g., secure to avoid predators and/or extreme weather) and the distance to a foraging area^13^. Multiscale studies allow identifying important scales concerning individual perception of their habitats, otherwise it is difficult to detect when knowledge of the ecology of the study species is limited^14^.

The White-throated Treerunner (*Pygarrhichas albogularis* King 1831; Furnariidae), is a poorly known passerine endemic to South American temperate and Mediterranean ecosystems, mainly found in central and southern regions of Chile and Argentina^15–17^. This species is considered an “old relict” as it is the only living species of the genus *Pygarrhichas*^18,19^, and according to the International Union for the Conservation of Nature their populations are decreasing^20^. White-throated Treerunner is one of the four species of tree cavity excavators in these ecosystems^21^. As an excavator, this species relies on habitats with presence of trees suitable for excavation^22^. Although this species has been suggested as a key habitat facilitator for several avian and mammalian cavity-nesting species in southern South America^16,21^, there are only two studies on the ecology of Treerunners and these focused on foraging use of tree species^23^ and its morphological description^15^. However, there are occasional community level studies that included Treerunners as part of an avian assemblage^24–32^.

In this study we examine the nesting preference of Treerunners and whether their preferred habitat attributes are linked to fitness through a two-step modelling approach. First, we assess habitat preferences analyzing the link between habitat attributes and preferences of nesting sites at: (1) nest-tree scale, (2) forest-stand scale, and (3) landscape scale. Second, we assess whether habitat attributes of sites used to breed are linked to fitness (represented as nest survival and number of fledglings) at the same three spatial scales, adding cavity characteristics as a fourth smaller spatial scale in the analysis. We hypothesize that (1) habitat attributes that are used to breed within a given landscape differ from the most available attributes and that (2) habitat attributes at each spatial scale improve fitness, and (3) there is a match between attributes of habitat used and fitness. This study provides a better understanding of forest attributes that must be maintained to ensure habitat and breeding success for this and other coexisting cavity nesting species in South American temperate forests^31^.

## Results

Between 2010 and 2018, we located and monitored 65 White-throated Treerunner nests (273 to 1,342 m of elevation); most of them were in freshly excavated cavities in *Nothofagus* trees with a broad range of DBH (Table 1). Old dead trees with advanced decay (55%) and live unhealthy trees (35%) contained the great majority of its nests, with only 8% and 2% for recently dead and live healthy trees, respectively. When we look at the specific substrate decay, 80% of the nests were in old dead substrates, and only 14% and 6% in live unhealthy and recently dead substrates, respectively. White-throated Treerunners laid an average of 3.2 ± 0.8 eggs (± standard deviation; range: 1–5 eggs). Laying, incubation and nestling periods were 4, 13 and 21 days on average, respectively. The daily survival rate was 0.9956, with an overall nest survival of 85%. Regarding failed nests (*n* = 10), 6 failed because of predation, 3 were abandoned, and 1 nest failed because eggs were not viable.

**Table 1:** Characteristics of nesting habitats (cavities, tree, forest-stand, and landscape scales) and fitness (measured as nest survival and the number of fledglings) of White-throated Treerunners (*Pygarrhychas albogularis*; *n* = 65 nests) in Andean temperate forests, Chile. Decay class 1, 2, 3 and 4 represent live healthy trees, live unhealthy trees, recently dead trees, and long dead trees, respectively.

Variable	White-throated Treerunners’ nests
Landscape scale	
Forest edge (m)	482.1 ± 807.7 (0–2851.8)
Forest area (m^2^)	25882.1 ± 4403.4 (9880–29499)
Forest-stand scale	
Tree density (tree/ha)	771 ± 366 (25–1550)
Average DBH (cm)	31.4 ± 7.7 (20.2–59.8)
Standard deviation DBH (cm)	16.2 ± 11.2 (6.9–86)
Decay class mode	2 ± 0.6 (1–4)
Tree scale	
Tree species (%)	*Nothofagus obliqua*: 61.5
	*Nothofagus dombeyi*: 12.3
	*Nothofagus pumilio*: 10.8
	*Persea lingue*: 9.2
	*Eucryphia cordifolia*: 6.1
Tree DBH (cm)	40.6 ± 20.3 (14.1–123.1)
Decay class	3.2 ± 1.0 (1–4)
Cavity scale	
Fresh (%)	Fresh cavities: 89.2
	Non-fresh cavities: 10.8
Entrance diameter (cm)	3.8 ± 0.6 (2.5–5)
DCH (cm)	21.5 ± 11.5 (9–60)
Height above ground (m)	9.6 ± 4.1 (1.1–17.1)
Aspect (°)	145.4 ± 106.5 (4–348)
Cavity volume (cm^3^)	1,349.1 ± 755.2 (147.3–4,417.9)
Branch order (%)	Main trunk: 64.6
	2nd order branch: 30.8
	3rd order branch: 4.6
Substrate decay class	3.7 ± 0.7 (2–4)
Fitness	
Nest survival (%)	85.04 (CI = 57.27– 98.16)
Number of fledglings (# chicks)	3.0 ± 1.2 (1–5)

Values are presented as percentages or mean ± standard deviation (range). CI means confidence intervals.

### Breeding habitat preferences across spatial scales

**Fig. 1:**
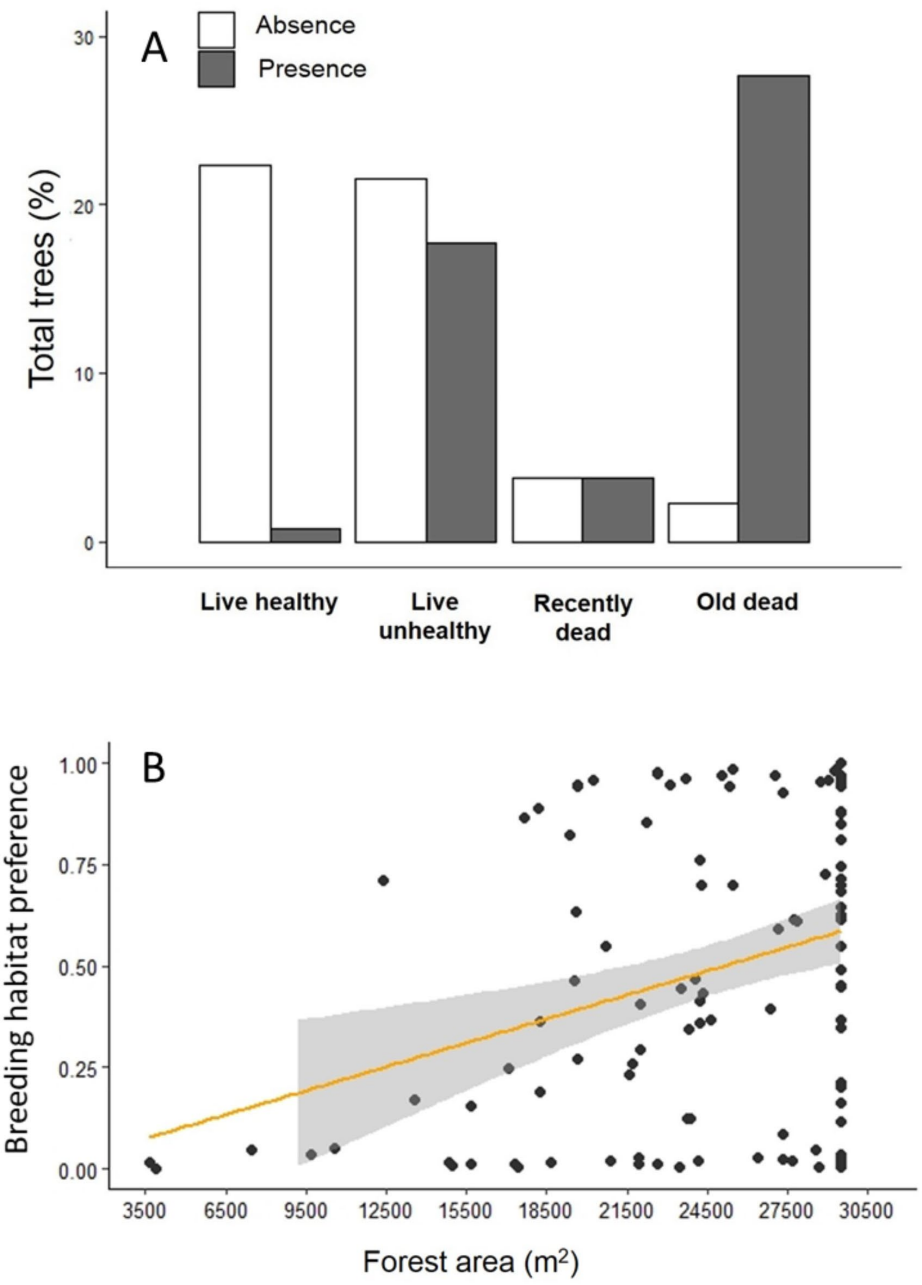
Most influential variables, at different spatial scales, on nesting habitat preferences of White-throated Treerunners (*Pygarrhichas albogularis*) in Andean temperate forests of South America: (**A**) Number of trees within each tree-decay class with presence or absence of nests (tree scale) and (**B**) forest area (landscape scale).

Variation in the use of breeding habitat was best predicted by a model that included two nest-tree scale variables (DBH and tree decay), one forest-stand scale variable (basal area; *b =* 4.64 ± 3.04, *p* > 0.05), and one landscape scale variable (forest area; Table 2). The probability that a given habitat is used for nesting increased with larger trees, advanced tree decay, and forest area. Following the b coefficients, tree decay was the strongest predictor of nest presence, followed by forest area and DBH. Treerunners avoided healthy living trees (*b =* -10.10 ± 2.86, *p* < 0.01), and the probability of nesting in a tree increased positively with each increasing decay class (Fig. 1A): unhealthy living trees (*b =* 3.53 ± 1.26, *p* < 0.01), recently dead trees (*b =* 3.76 ± 1.46, *p* = 0.01), and old dead trees (*b =* 7.35 ± 1.64, *p* < 0.01). Even when both forest area and DBH were positively associated with preferred breeding habitats, GLMMs showed that the extension of forest area (*b =* 4.09 ± 2.11, *p* = 0.05, marginally significant; Fig. 1B) influenced 82 times more than DBH (*b =* 0.04 ± 0.02, *p* = 0.01). This model provided a good fit for the data ($\:{\chi\:}^{2}$= 83.63, *p* = 0.99). The control values at the landscape, forest-stand, and tree scales can be found in the Supplementary Material 1.

**Table 2:** Model rankings for White-throated Treerunners (*Pygarrhychas albogularis*) habitat preference, number of fledglings, and nest survival in relation to the four spatial scales assessed in its nesting sites in South Andean temperate forests, Chile.

Model	K^a^	AICc	∆AIC^b^	W_i_^c^	LL^d^
Habitat preference					
DBH + Decay + Basal area + Forest area	**9**	**115.2**	**0.00**	**0.34**	**-47.8**
DBH + Decay + Basal area + Forest area + SD DBH	10	117.3	2.06	0.12	-47.67
Nest survival					
Entrance diameter + forest edge + height	**6**	**95.1**	**0.00**	**0.07**	**-41.44**
Entrance diameter + height	5	95.4	0.26	0.06	-42.61
Forest edge + height	5	95.9	0.81	0.05	-42.88
Height	4	96.1	0.98	0.05	-44.00
DBH + entrance diameter	5	96.4	1.27	0.04	-43.11
Entrance diameter + forest edge + height + tree density	7	96.7	1.77	0.03	-41.29
Entrance diameter + DBH + height	6	97.0	1.84	0.029	-42.36
Forest edge + height + tree density	6	97.0	1.92	0.03	-42.40
DBH	4	91.1	1.97	0.03	-44.49
DBH + Forest edge + height + entrance diameter	7	97.3	2.06	0.03	-41.43
Number of fledglings					
Decay class + aspect	**7**	**98.1**	**0.00**	**0.32**	**-40.23**
DBH + decay class + aspect	8	98.2	0.14	0.30	-38.71
Aspect	6	98.5	0.42	0.26	-43.34
Decay class + forest area + aspect	8	100.1	2.07	0.11	-39.67

Season and site were random terms in all models. Bold indicates best-supported models. ^a^ Number of parameters estimated. ^b^ Difference in AICc values between each model and the lowest AICc model (we show the list of models until the first one with ∆AIC > 2). ^c^ AICc model weight. ^d^ Log likelihood.

### Fitness outputs

**Fig. 2:**
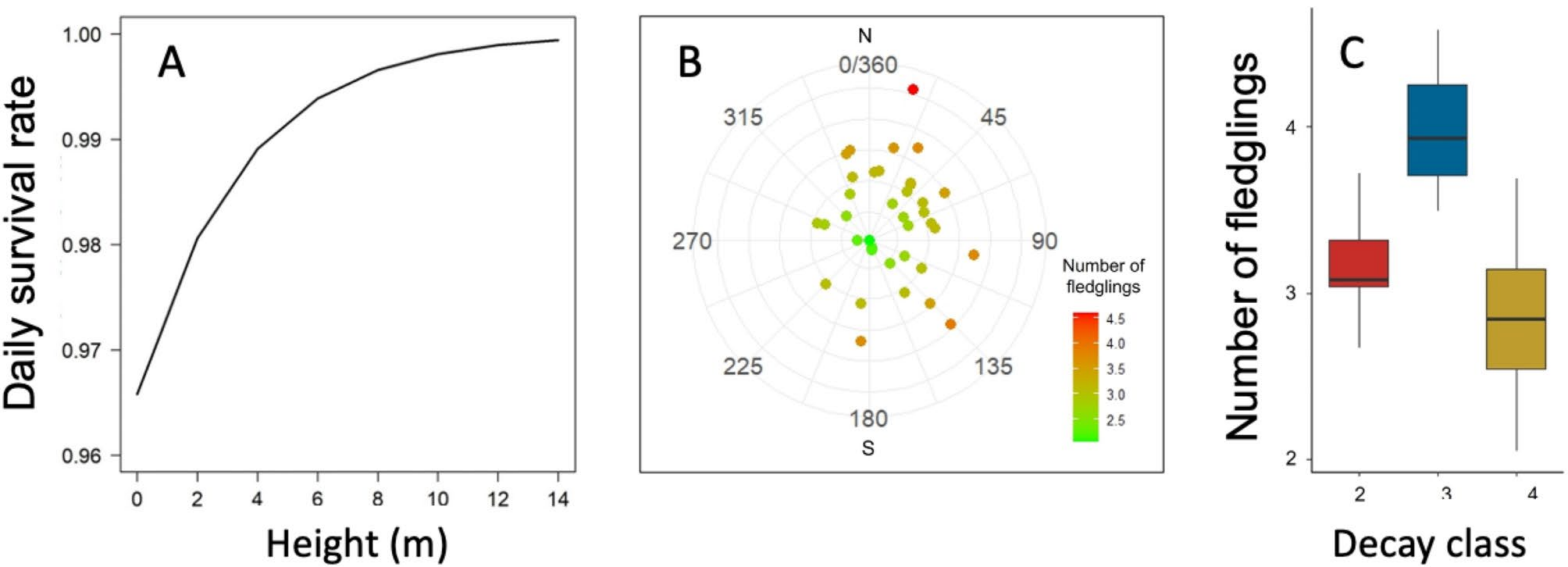
Most influential variables (significant and marginally significant), at different spatial scales, on fitness (daily survival rate and number of fledglings) of White-throated Treerunners (*Pygarrhichas albogularis*) in Andean temperate forests, South America: (**A**) Height above ground (cavity scale), (**B**) cavity orientation and number of fledglings (cavity scale), and (**C**) decay class (tree scale). Decay class 2, 3 and 4 represent live unhealthy trees, recently dead trees, and long dead trees, respectively.

For nest survival, there were two spatial scales included in the best models: cavity scale (entrance diameter and height) and landscape scale (forest edge, Table 2). Entrance diameter (*b* = 0.73, CI = −0.11–1.93, *p* = 0.13) and forest edge (*b* = 1.01, CI = −0.24–3.73, *p* = 0.28) had a positive effect on nest survival (daily survival rate). Nests with bigger entrances and further from the forest edge were more successful. Height was positively and marginally associated with nest survival (*b =* 1.09, CI = 0.24–2.84, *p* = 0.05; Fig. 2A). This model was a good fit for the data ($\:{\chi\:}^{2}$ = 151.92, *p* = 1.00).

Regarding the number of fledglings produced, the final model had parameters at the cavity (aspect) and tree (decay class) scales. Aspect had an important effect on number of fledglings produced, with nests facing south producing more nestlings (*b* = −0.39, CI = −0.62–0.14, *p* = 0.002; Fig. 2B). Tree decay also had an effect, with number of nestlings produced positively associated with unhealthy living trees (*b* = 3.02, CI = 2.48–3.46, *p* = < 0.001) and with recently dead trees (*b* = 0.43, CI = −0.42–3.46, *p* = 0.32), while long dead trees were negatively associated with the number of fledglings (*b* = −0.47, CI = −0.96–0.04, *p* = 0.05) (Fig. 2C). This model provided a good fit for the data ($\:{\chi\:}^{2}$ = 13.96, *p* = 0.99).

## Discussion

We found that both habitat preferences for nesting and nest survival measures for the White-throated Treerunner operate across multiple spatial scales. Similar to previous studies using multiscale approaches^7,13,33^, at least one variable at each scale influenced nest site preferences and/or fitness (number of fledglings and nest survival) for our study species. Variables at both tree and landscape scales were the most important influencing nesting habitat preferences, while variables at both cavity and tree scales were the most important in our assessment of fitness (nest survival and number of fledglings). The White-throated Treerunner showed preferences for areas with a relatively high forest cover and its nest survival increased with the distance from the forest edge. This may suggest that old-growth forests, with extensive areas of forest far from the edges, and with relatively less, but larger, trees compared to second-growth forests^34^, contain the best complement of multiscale variables for this species.

The White-throated Treerunner tends to excavate new cavities (89%) every nesting season, with the remaining 11% consisting of reused cavities, all of which were excavated by this species during previous years. Thus, we have determined that this species is a primary cavity-nester (i.e. obligate excavator; mainly excavates its own cavities, but occasionally uses existing excavated cavities)^35^. Breeding pairs might use existing cavities depending on nest predation risk, for instance, in case their breeding attempt failed in their fresh cavity. This pattern is similar to that found in a medium-sized woodpecker in North America, the Red-naped Sapsucker (*Sphyrapicus nuchalis*), which mostly excavate its own cavities, but about 11–12% of breeding pairs reuse existing cavities excavated in previous years^36^. Although the White-throated Treerunner has been compared to nuthatches in North America; the Red-Breasted nuthatch (*Sitta canadensis*) tends to be a facultative excavator (i.e. excavate about 50% of their nests, excavation rates vary annually;^37,38^). Thus, based on our study, Treerunners are more extensively primary cavity excavators resembling more Red-naped Sapsuckers or Downy Woodpeckers (*Dryobates pubescens*) rather than nuthatches that are facultative excavators^22^.

White-throated Treerunner nesting habitat preferences were associated mainly with variables acting at both tree and landscape scales, preferring relatively larger trees in advanced decay classes present in highly forested areas. This partially agrees with the results found for functionally similar species in the northern hemisphere, such as the Red-naped Sapsucker^13^. The latter species prefers nesting habitats associated with landscape scale attributes, similar to Treerunners; however, only tree scale attributes are linked to their fitness^13^. This suggests a hierarchical process of habitat selection for the White-throated Treerunner^33^, in which several factors, at different spatial scales, influence the decision of choosing a given nest-site^7^. For example, the main trophic resource for Treerunners is forest-dwelling arthropods in adult and larval states^39,40^, which are probably more abundant in multi-stratified forested landscapes with presence of all tree decay classes^16^. Additionally, this species is choosing nest site variables at a finer scale. Like other cavity-nesting species in the study area^21^, tree scale attributes (especially tree decay) are the most influential factors operating in nesting habitat selection for Treerunners. This finding is similar to results reported for several primary cavity nesters such as woodpeckers in temperate forests of North America^22^ and in tropical Atlantic forests of South America^41^, where excavators strongly select live unhealthy and standing dead trees as nesting sites^7,42^.

The White-throated Treerunner showed 85% nest survival. This high survival rate strongly contrasts with the overall nest survival of avian communities in tropical forests of Brazil (42%) and Dominican Republic (34%)^44^, but is similar to cavity nesters and excavator species inhabiting other temperate forests such as Red-Breasted nuthatches (84%) and Red-naped Sapsuckers (91%)^13^. We found that nest survival was positively associated with the entrance diameter of cavities, a counterintuitive finding, which might be associated with adult ability to successfully breed; and thus, stronger adults might be able to protect the nest and excavate larger cavities. Furthermore, cavity-nest predator assemblage in our study area is diverse but composed by relatively large animals, with the mammals *Leopardus guigna*, *Dromiciops gliroides*, and *Rattus rattus*, and the birds *Milvago chimango*,* Caracara plancus*,* Glaucidium nana*, being the main predators^46^. Thus, even when predation was the main cause of nest failure, small differences in entrance diameter may not increase predation risk. On the other hand, excavated cavities higher in the tree might reduce the predation risk by terrestrial predators. Nest survival increased with the distance from the forest edge, suggesting that the selection of breeding habitats (selecting sites with high forest cover) is adaptive, and this decision is being translated into higher nest survival^13^. Second-growth forests contain a larger number of trees and more chance to be close to the edge compared to old-growth forests, as well as fewer options to nest higher in the canopy. This suggests that second-growth forests may not provide ideal breeding habitats for White-throated Treerunners.

The number of fledglings of White-throated Treerunners was associated with cavity and tree scale attributes (i.e. aspect and decay). This pattern is similar to the productivity of Red-naped Sapsuckers in the United States^13^, where cavity and tree scales were also the most important attributes associated with the number of fledglings. However, breeding outputs were positively related to southeastern orientation (facing the equator in the Northern Hemisphere) of cavity entrance for Red-naped Sapsuckers, while we found that south-orientated cavities (away from the equator in the Southern Hemisphere) produced more nestlings for Treerunners. Our focal species excavated cavities facing almost all cardinal directions, agreeing with recently published results for South American excavators, in which there is no general pattern of cavity orientation^47^. This finding may represent a slight mismatch between use and fitness of cavity orientation, suggesting that other site-specific variables, such us slope orientation, might be influencing the aspect of the Treerunners’ cavities^47^. Instead, and unlike nest survival, the number of fledglings was associated with tree scale, being associated with tree decay. We found that unhealthy live trees and recently dead trees strongly increased the number of fledglings (a match with their preference), while long dead trees negatively affected the number of fledglings (a mismatch with their preference). This might be also responding to adult ability to generate more breeding output; perhaps stronger adults select stronger substrates to excavate, leaving softer trees and branches for the weaker pairs. However, it is also possible that factors not measured in this study, but probably related to tree decay, are affecting breeding productivity.

Cavity-nesters globally have become seriously threatened by deforestation^48^ and conventional silviculture^41,49,50^, in which old and large cavity-bearing trees are often removed. This might also be the case for White-throated Treerunners, and for other cavity-nesting species that nest in large decaying and standing dead trees in south temperate forests^21^. Despite their importance as cavity substrates, large decaying and standing dead trees are not protected by current forestry laws in Chile^29^. Here, temperate forests are threatened by fragmentation, degradation, and deforestation^51,52^, as nearly 70% of their original extent has been lost^53^. Thus, and following the actions proposed by the Chilean Bird Conservation Strategy^54^, we call for a sustainable management to maintain the important forest attributes at different scales (especially both live unhealthy and recently dead trees, and forest cover), for Treerunners and the persistence of the cavity-nesting community. Our reported relationship between habitat preferences and fitness could be crucial information to conserve White-throated Treerunners and multiple coexisting species, as this excavator is known to play an important role structuring forest-dependent communities by providing cavities for other small-size vertebrates, including birds, marsupials, and bats^21,30^.

## Methods

### Study area and focal species

We studied White-throated Treerunners in Andean temperate forests of La Araucanía Region, Chile (39°16′S, 71°48′W, see^21^ for a full description of the study area). This area presents a mean daily temperature of 6.0 °C and an average annual precipitation > 2,000 mm distributed throughout the year^30,55^. We surveyed 20 forest sites (20–40 ha each), corresponding to nine second-growth forests between 40 and 80 years old subjected to selective logging, and 11 old-growth forests over 200 years old with less anthropogenic pressure. Second-growth forests are dominated by broadleaf species such as *Nothofagus obliqua*, *Nothofagus dombeyi*, and *Laurelia sempervirens*, while old-growth forests were mixed conifer-broadleaf forests dominated by broadleaf species such as *Laureliopsis philippiana* and *Nothofagus dombeyi* associated with the conifer *Saxegothaea conspicua* at lower elevations (500–900 m). At higher elevations (900-1,600 m), old-growth forests include *Nothofagus pumilio* (broadleaf) and *Araucaria araucana* (conifer). The understory of second- and old-growth forests were dominated by bamboo species (*Chusquea* spp.), *Azara* spp., *Berberis* spp., and tree saplings.

In Chile, the White-throated Treerunner is distributed between Santa Inés hill (32° 9′31.51″S; 71°29′32.76″W) and the Cape Horn Archipelago (55°58′59.61″ S; 67°16′00.69″ W^15,17^. Because of their size, morphology, and habits, White-throated Treerunners have been compared to “Nuthatches” (Sittidae, genus *Sitta*) from North America, Europe, and Asia^56,57^. The White-throated Treerunner is strictly arboreal, does not fly long distances, and similar to nuthatches moves from tree to tree, climbing them vertically with its legs and tail^18,57^. It actively feeds on larvae, adult insects^17,58^ and other arthropods^40^, by removing small pieces of bark along tree trunks and branches^17,18^. At early stages of the reproductive season, naturalist reports indicate that the Treerunner excavates its nesting-cavities on highly decayed or burned trees^57,58^ and standing dead trees of small diameter at breast height (15 cm)^58^.

### Nest searching and monitoring

During eight breeding seasons (October to February), between 2010 and 2018, we searched (6 h per day, 6 days per week) for occupied cavities of White-throated Treerunner in each of the 20 forests. To find and monitor nests, we employed the protocol described in Martin and Geupel (1993), observing adult behavior such as repeated visits to the same tree, long periods out of sight after knowing its position on a tree or sudden flight out of a tree-cavity^60^.

Nests in cavities lower than 2 m height were checked directly using a Ridgid camera, while a wireless monitoring system^61^ mounted on a 15 m long telescopic pole^21,41,60^ was used for cavities above 2 m height. A nest was considered active after we confirmed it contained at least one egg or nestling. Then, we obtained geographic coordinates for each nest using a handheld GPS with ± 10 m accuracy. A unique code was given to every nest, cavity, and tree containing a nest. Each of them, was monitored every 3–4 days until the nest attempt ended (either failed or successful) to determine number of hatched eggs and fledglings, signs of depredation, or cavity availability for further nesting attempts.

### Habitat sampling across spatial scales

**Fig. 3:**
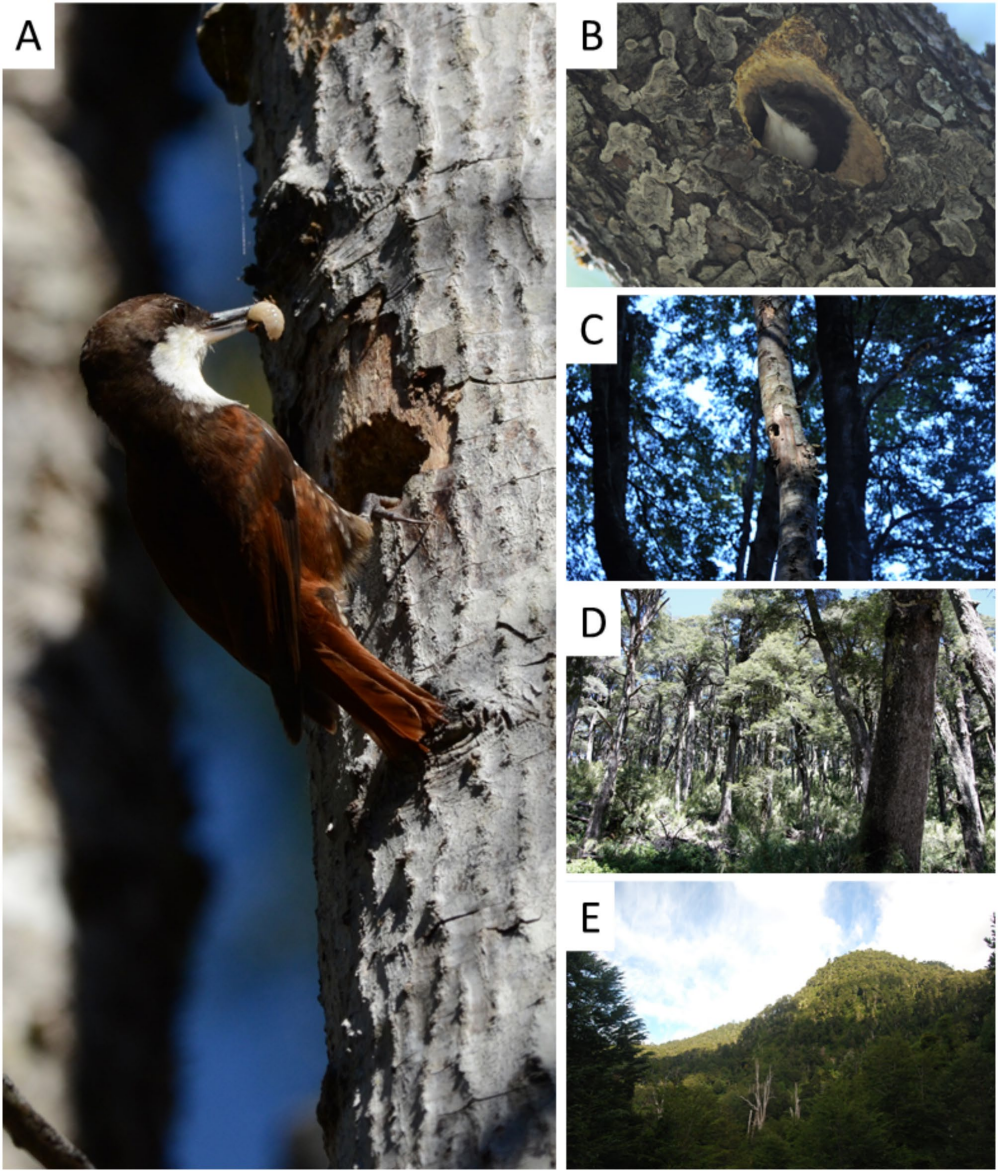
Study species White-throated Treerunners (*Pygarrhychas albogularis*) (**A**) and the spatial scales assessed for breeding habitat preferences and fitness in Andean temperate forests of South America: cavity scale (**B**), tree scale (**C**), forest-stand scale (**D**), and landscape scale (**E**). Photos were taken by Tomás A. Altamirano (A, C, D), Fernando J. Novoa (B), and J.Tomás Ibarra (D).

Following Sadoti and Vierling (2010), we used a paired used-availability study design to infer habitat preference at three spatial scales (tree, forest-stand, and landscape) and their link to fitness at four spatial scales (cavity, tree, forest-stand, and landscape; Table 3; Fig. 3). At (1) cavity-scale, we measured entrance diameter (cm), diameter at cavity height (DCH, m), cavity height above the ground (m), aspect (°), internal cavity volume (cylinder dimension, cm^3^), branch order (order of tree branches where excavated cavity was located; 1: main trunk; 2 secondary branch; and 3: tertiary branch), and substrate decay class (degree of decomposition of the specific substrate where a given nest was located, associated with the branch order). At (2) nest-tree scale, we recorded tree species, diameter at breast height (DBH), and decay class of nest-trees (1: live healthy tree; 2: live unhealthy tree; 3: recently dead tree; 4: old dead tree; and 5: naturally fallen tree; for details see^21^; a detailed protocol can be found in Supplementary Material 2). At (3) forest-stand scale, we established vegetation plots of 11.2 m radius (0.04 ha), with the nest-tree at the center of the plot and recorded both DBH and decay classes for every tree with DBH > 12.5, because it is the minimum diameter that can support a Treerunner nest^21^. These data allowed us to calculate habitat attributes including tree density, mean DBH and standard deviation, decay class mode, and stand basal area^14^. At (4) landscape scale, we used a 3 ha buffer for analysis with nest-trees at the center. We choose a 3 ha buffer because other studies reported home ranges of 3 ha for Brown-headed nuthatch (*Sitta pusilla*)^62^ and Eurasian nuthatch (*Sitta europaea*)^63^; species of comparable size and habits to the White-throated Treerunner. At this scale, we measured the nearest distance to a forest edge and forest area^14^ (Table 3). We determined forest cover area through remote sensing combining Remap^64^ and QGIS 3.6 Noosa^65^. Remap is an online mapping platform that allowed us to classify land cover. At an approximate 120.000 ha buffer area containing all 130 plots (65 corresponding to White-throated Treerunner nests and 65 to control trees, i.e. random trees without nests of the study species), we established a set of training points through photointerpretation. This set of training points feeds an algorithm that based on a set of biophysical, spectral, and climatic predictors classifies the entire area in the different classes of land cover types that were defined with the training set points. The image of reference corresponded to a 2014–2017 Landsat composite image. The resulting classified image was downloaded and corrected with QGIS, determining the forest cover at 3 ha plots for every nest and control. As Eurasian nuthatch shows a maximum distance of 50 m in open spaces^66^, we established and measured forest edge when there was a > 50 m distance between forest patches^69^.

**Table 3:** Spatial scales (cavity, nest-tree, forest-stand and landscape) assessed to explore habitat preference and fitness of White-throated Treerunners (*Pygarrhychas albogularis*) in Andean temperate forests, Chile.

Spatial scale	Variable	Description
Cavity	Fresh	1: Cavity excavated during the observation year; 0: Cavity excavated in previous seasons
	Entrance diameter	Horizontal diameter of the entrance of the excavated cavities (cm)
	DCH	Diameter at cavity height of nest trees (cm)
	Height	Height above the ground of the excavated cavities (m)
	Aspect	Cardinal orientations of the excavated cavities in degrees (0° − 360°)
	Cavity volume	Internal cavity volume calculated as volume of a cylinder (cm^3^)
	Branch order	Order of tree branches where excavated cavity was located. 1: main trunk; 2 secondary branch; 3: tertiary branch
	Subdecay class	Degree of decomposition of the tree branch where excavated cavity was located
Nest-tree	Tree species	Tree species where nests were found
	Tree DBH	Diameter at breast height of nest trees
	Decay class	Degree of decomposition of the nest trees. Decay classes assigned were 1 (live healthy tree); 2 (live unhealthy tree); 3 (recently dead tree); and 4 (long dead tree; modified from^75,76^
Forest-stand	Tree density	Density of total trees in one hectare
	Average DBH	Mean DBH of total trees in forest-stand
	Standard deviation DBH	Standard deviation of DBH in total trees on forest-stand
	Decay class mode	Mode of degree of decomposition of the total trees in forest-stand
	Basal area	Basal area of forest-stand (cm^2^/ha)
Landscape	Forest edge	Nearest distance to forest edge from nest tree (m).
	Forest area	Forest area in circular buffer of 3 ha with nest tree in the center (m^2^)

### Statistical analysis

#### Breeding habitat preferences

We used a stratified case-control sampling design^67^ to examine habitat attributes, across spatial scales, associated with nest-site preferences and fitness. We assume breeding attempts to be independent, regardless of breeding pair identity. We had 65 nests of the White-throated Treerunner, at a minimum average distance of 0.4 km, and thus randomly selected 65 control sites from a large data base we have collected throughout the years (2008–2018). For the random selection of control sites, we excluded plots where Treerunner nests were found and we selected control vegetation plots and trees from the same forest site and season of its paired Treerunner nest.

To examine habitat preferences, we used generalized linear mixed effect models with a binomial distribution family (R package lme4 v1.1-31)^68^, and with site and season as random effects, to control for any inherent capacity of a given forest site or season to support more nests, and White-throated Treerunner nest presence (1) or absence (0) as the response. We assessed possible preferences at three spatial scales (nest-tree scale, forest-stand scale, and landscape scale, Table 3). The modelling algorithm was considered keeping all random effect variables as a basis, testing the 11 single models first (including the Null Model), and then manually adding fixed effect variables that were significant, one at a time from smaller to larger scales. Twenty-three models were built, and the best model was selected through Akaike criterion (i.e. the most parsimonious model given by the lower ΔAIC value) and variable significance. Coefficients are shown ± their standard errors.

#### Fitness

To investigate whether habitat attributes of used reproduction sites are linked to fitness we looked at two different aspects: nest survival and number of fledglings. For nest survival we estimated daily nest survival rate (DSR) using the logistic exposure method^69^ with generalized linear mixed effect models (binomial distribution family, with the link function code adapted from^69^; Supplementary Material 3), including site and season as random effects. The response variable was either 1 (nest survived between nest visits) or 0 (nest did not survive the interval between visits). For the number of fledglings produced, we used linear mixed effect models, with a Poisson distribution family, and with number of fledglings as the response variable and included site and season as random effects. As for the fixed effects, we used the same three spatial scales used in the habitat preference assessment, but also added cavity-scale variables in the analysis (Table 3). This was done only for fitness and not habitat selection because, as an excavator, cavity attributes are generated by the excavation process; thus, those attributes are not “preferred” or “avoided”. We fitted all possible combinations of variables, excluding interactions. For most variables we looked at their linear effect only. However, for aspect, as it is a circular variable and we are interested in contrasting north vs. south exposed cavities, we transformed it into “northness” (cos(aspect)), which ranges from − 1 (south-oriented cavities) and 1 (north-oriented cavities)^70,71^. We then ranked the models by AICc and selected the model with the lowest value. We assessed parameter importance in the final model by determining whether or not their 95% confidence interval (CI) included zero^72^.

We estimated DSR of the population with the intercept of the null DSR model. To estimate overall survival, we raised DSR to an exponent of 38 which represents the average duration of the whole nesting cycle for this species in this area (laying = 4 days [assuming 1 egg laid per day], incubation = 13 days, nestling = 21 days). Before fitting nest survival models we investigated a potential effect of researcher on DSR derived from frequent nest visitations. We created a continuous variable of cumulative nest visitations, and assessed its effect on DSR using logistic exposure method, as described above. Before fitting any model, we checked for outliers with Cook’s distance (D), and for correlation among covariates to assess multicollinearity (*r* > 0.75); there were no outliers and no correlations in the analysis. We replaced missing values with the mean of the variable and standardized all continuous variables to a mean of zero with one unit of standard deviation^73^. We assessed the goodness of fit of the final models with $\:{\chi\:}^{2}$ tests, rejecting the model if *p* < 0.05. All analysis were performed in R 4.2.1 ^74^.

## Electronic supplementary material

Below is the link to the electronic supplementary material.

Supplementary Material 1

Supplementary Material 2

Supplementary Material 3

## Data Availability

The data that support the findings of this study are available from the corresponding author upon reasonable request.
