# Supplementary material for: Linking habitat preferences and fitness across scales for a relict bird species of the southern Andes
Source: Sci Rep. 2025 Oct 6;15:34667. doi: 10.1038/s41598-025-93594-1 (PMC12501267; doi:10.1038/s41598-025-93594-1)
Supplement: Supplementary file 1 — Supplementary Material 1 [file 41598_2025_93594_MOESM1_ESM.docx]

**LINKING HABITAT PREFERENCES AND FITNESS ACROSS SCALES FOR A RELICT BIRD SPECIES OF THE SOUTHERN ANDES**

**short runnung title: Habitat preferences and FITNESS of TREERUNNERS**

Tomás A. Altamirano^1,2,3,4*^, Fernando J. Novoa^4,5^, Zoltan Von Bernath^5^, Alejandra Vermehren^5^, Kathy Martin^3,6^, Rocío Jara^4,5^, Edwin Rockwell Price^7^, Ricardo Rozzi^4,8,9^ & José Tomás Ibarra^4,5,10^

Suppememntary Material 3. Adapted code for the nest survival rate from Shaffer 2004.

**"logexp <- function(exposure = 1)**

**{**

**linkfun <- function(mu) qlogis(mu^(1/exposure))**

**linkinv <- function(eta) plogis(eta)^exposure**

**logit_mu_eta <- function(eta) {**

**ifelse(abs(eta)>30,.Machine$double.eps,**

**exp(eta)/(1+exp(eta))^2)**

**}**

**mu.eta <- function(eta) {**

**exposure * plogis(eta)^(exposure-1) ***

**logit_mu_eta(eta)**

**}**

**valideta <- function(eta) TRUE**

**link <- paste("logexp(", deparse(substitute(exposure)), ")",**

**sep="")**

**structure(list(linkfun = linkfun, linkinv = linkinv,**

**mu.eta = mu.eta, valideta = valideta,**

**name = link),**

**class = "link-glm")**

**}”**
