# Supplementary material for: Linking habitat preferences and fitness across scales for a relict bird species of the southern Andes
Source: Sci Rep. 2025 Oct 6;15:34667. doi: 10.1038/s41598-025-93594-1 (PMC12501267; doi:10.1038/s41598-025-93594-1)
Supplement: Supplementary file 2 — Supplementary Material 2 [file 41598_2025_93594_MOESM2_ESM.docx]

**LINKING HABITAT PREFERENCES AND FITNESS ACROSS SCALES FOR A RELICT BIRD SPECIES OF THE SOUTHERN ANDES**

**short runnung title: Habitat preferences and FITNESS of TREERUNNERS**

Tomás A. Altamirano^1,2,3,4*^, Fernando J. Novoa^4,5^, Zoltan Von Bernath^5^, Alejandra Vermehren^5^, Kathy Martin^3,6^, Rocío Jara^4,5^, Edwin Rockwell Price^7^, Ricardo Rozzi^4,8,9^ & José Tomás Ibarra^4,5,10^

**Supplementary Material 1.** Variables of the random control trees at the three scales: landscape, forest-stand and tree.

| **Variable** | **Control nests** |
| --- | --- |
| *Landscape scale* | |
| Forest edge (m) | 5003.9 ± 8486 (0 - 28304) |
| Forest area (m^2^) | 23508 ± 6918 (3647- 29499) |
| *Forest-stand scale* | |
| Tree density (tree/ha) | 723 ± 355.9 (100 - 1750) |
| Average DBH (cm) | 30.7 ± 6.7 (20 - 51.5) |
| Standard deviation DBH (cm) | 15.6 ± 6 (3.1 – 31.1) |
| Decay class mode | 2 ± 0.7 (1 - 4) |
| *Tree scale* | |
| Tree species (%) | *Gevuina avellana* : 26.2  Unknown: 21.5  *Nothofagus obliqua*: 24.6  *Nothofagus dombeyi*: 10.8  *Nothofagus pumilio*: 10.8  *Eucryphia cordifolia*: 4.6  *Persea lingue*: 1.5 |
| Tree DBH (cm) | 34.9 ± 19.6 (15 - 128.5) |
| Decay class | 1.7 ± 0.8 (1 – 4) |
