# Supplementary material for: Linking habitat preferences and fitness across scales for a relict bird species of the southern Andes
Source: Sci Rep. 2025 Oct 6;15:34667. doi: 10.1038/s41598-025-93594-1 (PMC12501267; doi:10.1038/s41598-025-93594-1)
Supplement: Supplementary file 3 — Supplementary Material 3 [file 41598_2025_93594_MOESM3_ESM.docx]

**LINKING HABITAT PREFERENCES AND FITNESS ACROSS SCALES FOR A RELICT BIRD SPECIES OF THE SOUTHERN ANDES**

**short running title: Habitat preferences and FITNESS of TREERUNNERS**

Tomás A. Altamirano^1,2,3,4*^, Fernando J. Novoa^4,5^, Zoltan Von Bernath^5^, Alejandra Vermehren^5^, Kathy Martin^3,6^, Rocío Jara^4,5^, Edwin Rockwell Price^7^, Ricardo Rozzi^4,8,9^ & José Tomás Ibarra^4,5,10^

**Supplementary Material 2.** Protocol to determine tree decay class.

| **Tree decay class** | **Sub clases** | **Explanation** |
| --- | --- | --- |
| 1 | A | Live and healthy tree. |
| 2 | B | Living with signs of decay, such as fungal presence, mechanical damage, and feeding activity from woodpeckers, sapsuckers, and borers. Less than 20% of standing branches are dead. |
|  | C | Alive with more advanced signs of decay. 21-80% of branches are dead. Broken top (BT). |
|  | D | Alive, although nearly dead. More than 81% of branches are dead. |
| 3 | E | Recently dead tree, with 100% of the branches dead but still intact (i.e. without fallen branches), stable. |
|  | F | Dead tree with significant dead branch loss/fall (approximately 50%), broken top (BT), less than 50% bark loss, mostly hard wood. |
| 4 | G | Long dead, nearly branchless, broken top (BT), more than 75% bark loss. Both hard and soft wood present. |
|  | H | Long dead, branchless, barkless, broken top (BT), and soft wood. |
| 5 | 7 | Naturally fallen tree (e.g. wind or snow). |
|  | 8 | Tree fallen by cutting. |
| UKN | UKN | Unknown or undetermine. |
